# The role of CD101-expressing CD4 T cells in HIV/SIV pathogenesis and persistence

**DOI:** 10.1371/journal.ppat.1010723

**Published:** 2022-07-22

**Authors:** Zachary Strongin, Timothy N. Hoang, Gregory K. Tharp, Andrew R. Rahmberg, Justin L. Harper, Kevin Nguyen, Lavinia Franchitti, Barbara Cervasi, Max Lee, Zhan Zhang, Eli A. Boritz, Guido Silvestri, Vincent C. Marconi, Steven E. Bosinger, Jason M. Brenchley, Deanna A. Kulpa, Mirko Paiardini

**Affiliations:** 1 Division of Microbiology and Immunology, Yerkes National Primate Research Center, Emory University; Atlanta, Georgia, United States of America; 2 Barrier Immunity Section, Laboratory of Viral Diseases, NIAID, NIH; Bethesda, Maryland, United States of America; 3 Flow Cytometry Core, Emory Vaccine Center, Emory University; Atlanta, Georgia, United States of America; 4 Vaccine Research Center, National Institutes of Health; Bethesda, Maryland, United States of America; 5 Department of Pathology and Laboratory Medicine, Emory University School of Medicine; Atlanta, Georgia, United States of America; 6 Division of Infectious Diseases, Emory University School of Medicine; Atlanta, Georgia, United States of America; 7 Division of Infectious Diseases Research, Atlanta Veterans Affairs Medical Center; Atlanta, Georgia, United States of America; 8 Rollins School of Public Health, Emory University; Atlanta, Georgia, United States of America; 9 Emory Vaccine Center, Atlanta, Georgia, United States of America; University of Wisconsin, UNITED STATES

## Abstract

Despite the advent of effective antiretroviral therapy (ART), human immunodeficiency virus (HIV) continues to pose major challenges, with extensive pathogenesis during acute and chronic infection prior to ART initiation and continued persistence in a reservoir of infected CD4 T cells during long-term ART. CD101 has recently been characterized to play an important role in CD4 Treg potency. Using the simian immunodeficiency virus (SIV) model of HIV infection in rhesus macaques, we characterized the role and kinetics of CD101^+^ CD4 T cells in longitudinal SIV infection. Phenotypic analyses and single-cell RNAseq profiling revealed that CD101 marked CD4 Tregs with high immunosuppressive potential, distinct from CD101^-^ Tregs, and these cells also were ideal target cells for HIV/SIV infection, with higher expression of CCR5 and α4β7 in the gut mucosa. Notably, during acute SIV infection, CD101^+^ CD4 T cells were preferentially depleted across all CD4 subsets when compared with their CD101^-^ counterpart, with a pronounced reduction within the Treg compartment, as well as significant depletion in mucosal tissue. Depletion of CD101^+^ CD4 was associated with increased viral burden in plasma and gut and elevated levels of inflammatory cytokines. While restored during long-term ART, the reconstituted CD101^+^ CD4 T cells display a phenotypic profile with high expression of inhibitory receptors (including PD-1 and CTLA-4), immunsuppressive cytokine production, and high levels of Ki-67, consistent with potential for homeostatic proliferation. Both the depletion of CD101^+^ cells and phenotypic profile of these cells found in the SIV model were confirmed in people with HIV on ART. Overall, these data suggest an important role for CD101-expressing CD4 T cells at all stages of HIV/SIV infection and a potential rationale for targeting CD101 to limit HIV pathogenesis and persistence, particularly at mucosal sites.

## Author summary

While much progress has been made in the field of HIV persistence, there remain a number of outstanding mechanistic questions about cells that critically contribute to HIV pathogenesis and the maintenance of the viral reservoir during antiretroviral therapy. In particular, there is a great interest in identyfing surface markers on cells that play a role in pathogenesis and persistence to elucidate potential therapeutic targets. In this manuscript, we describe CD101-expressing CD4 T cells as an immunosuppressive population that is preferentially depleted following SIV/HIV infection and find that this loss is associated with higher viral burden and increased inflammatory cytokine levels. Furthermore, during long-term antiretroviral therapy, these cells display a phenotypical and functional profile consistent with cells that may critically contribute to reservoir persistence, including high inhibitory receptor expression and production of immunosuppressive cytokines. Future studies of therapies modulating CD101 expression could uncover additional strategies for HIV therapeutics aimed at limiting residual inflammation and viral persistence.

## Introduction

Modern antiretroviral therapy (ART) is a critical tool for managing HIV infection, with high effectiveness at suppressing HIV replication and remarkable reduction of HIV morbidity and mortality [1,2]. However, due to the early establishment of a latent viral reservoir, HIV persists and, with very few exceptions, cessation of ART in people with HIV (PWH) results in rapid viral rebound and continued disease progression within weeks [3,4]. Therefore, defining key cellular contributors to HIV persistence remains critical to help define strategies to target and eliminate the reservoir in hopes for a functional cure.

With a recent focus in the field on the HIV reservoir, the characteristics of cells that contribute to this latent population have been well defined to this point. The reservoir has been shown to persist in a variety of CD4 T cell subsets, with critical contributions from central and effector memory cells, T follicular helper cells (TFh), Th17 cells and T regulatory cells (Tregs) [5–16]. Furthermore, the persistence of cells harboring HIV DNA has been linked to expression of a number of inhibitory receptors, including PD-1, TIGIT, LAG-3 and CTLA-4 [6,12,17,18]. Cells that critically contribute to long-term HIV persistence after ART initiation are also known to be preferentially infected during active HIV replication [7–9,19–21]. Furthermore, recent investigations have confirmed that the reservoir is primarily established at the time of ART initiation [22,23]. Finally, early ART initiation can reduce the chronic inflammation associated with increased morbidity/mortality risk during treated HIV infection, indicative of the importance of limiting acute HIV pathogenesis for long term health [24]. As a whole, these findings suggest that better understanding of target cell populations during early, active HIV infection, as well as continued description of characteristic markers of HIV persistence on ART, will inform the design of targeted therapeutic interventions aiming to reduce residual inflammation and the size of the viral reservoir.

The cellular features described above suggest an important role for negative regulators of T cell activation in favoring the persistence of HIV DNA in CD4+ T cells. Based on those findings, we investigated the potential role of CD101 in HIV pathogenesis and persistence. CD101 is a cell surface glycoprotein with an unknown ligand that is upregulated upon cell activation, inhibits T cell activation and proliferation, and critically regulates CD4 Tregs functions [25–29]. CD101^+^ Tregs are more suppressive than CD101^-^ Tregs *in vitro* and *in vivo*, and CD101 can be used to discriminate the functional potency of Tregs in mice [28,30]. Furthermore, CD101 has been described as a marker of resident-memory T cells and has been shown to be anti-inflammatory in gut mucosa [30–32]. In addition to its suppressive role on CD4 T cells, CD101 is specifically induced on terminally differentiated and highly dysfunctional CD8 T cells during chronic viral infection [33,34]. Overall, the association of CD101 expression with Tregs, gut mucosa, and inhibitory receptor expression, together with its anti-inflammatory role in gut mucosa, suggests a potential key role for CD101^+^ CD4 T cells in HIV pathogenesis and persistence.

In this study, we take advantage of the established SIV model of HIV infection in rhesus macaques (RMs), which allows for longitudinal analyses and tissue access, to investigate dynamics of CD101-expressing CD4 T cells in healthy, acutely-infected, and ART-suppressed RMs. We confirm high expression of CD101 on CD4 Tregs in healthy RMs, with distinguishing phenotypic and transcriptional signatures of higher suppressive capability compared with CD101^-^ Tregs. During acute SIV infection, CD101^+^ CD4 T cells are preferentially depleted as compared to their CD101^-^ counterpart, with depletion more pronounced for CD101^+^ Tregs and CD101^+^ CD4 T cells in the gut mucosa. This depletion is associated with higher viral burden in the gut, as well as higher plasma viral load and levels of inflammatory cytokines. After long-term ART, CD101^+^ CD4 T cells are restored to pre-infection levels, harbor intact SIV DNA at levels comparable to those found in the other memory CD4 T cells, and are enriched in inhibitory markers suggestive of a potential for long-term maintenance of the latent viral reservoir. These findings in RMs were extended and confirmed in a cohort of viremic and ART-suppressed PWH. Overall, while CD101^+^ cells were not directly enriched for HIV DNA, these findings reveal a preferential depletion of anti-inflammatory CD101^+^ CD4 T cells and suggest a critical role for CD101^+^ cells in HIV/SIV inflammation and in long-term viral persistence.

## Results

### CD101 is expressed on memory CD4 T cells and highly upregulated on regulatory CD4 T cells

**Fig 1:**
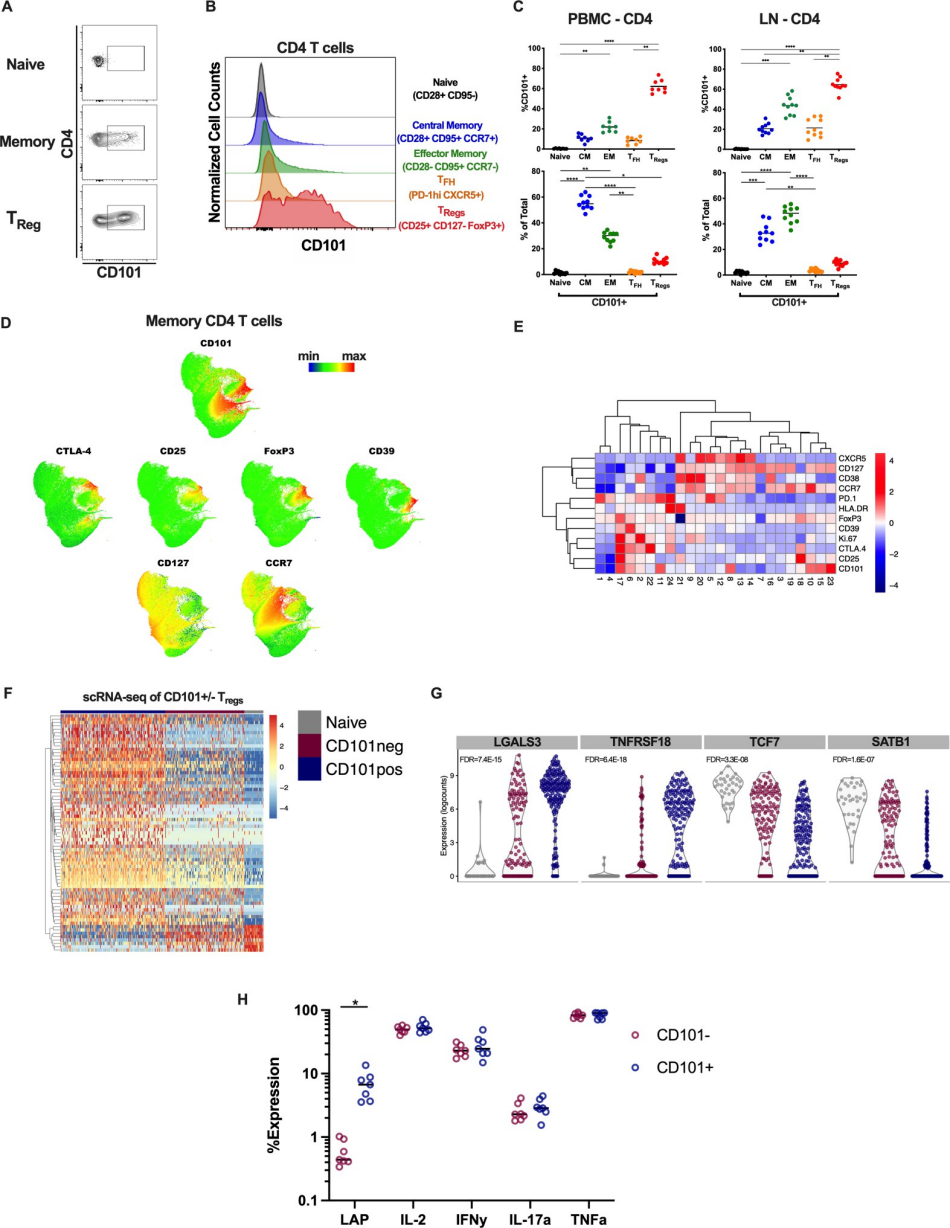
Phenotype of CD101-expressing CD4 T cells in healthy rhesus macaques. A) Representative staining of CD101 on CD4 subsets in healthy RM PBMC. B) Representative histograms of CD101 on detailed CD4 subsets in healthy RM PBMC. C) Expression of CD101 within detailed CD4 subsets in PBMC and LN from healthy RM (top); total contribution of CD101^+^ cells to the overall CD4 pool from each CD4 subset in PBMC and LN. D) UMAP plot of flow cytometry data showing overall Phenograph cluster location and expression intensity of markers of interest. E) Hierarchical clustering of expression (z-score) for selected markers from flow cytometry data in each Phenograph cluster. F) Heatmap of the top 50 differentially expressed genes between CD101^+^ and CD101^-^ Tregs (CD95+ CD25+ CD127-) by scRNA-seq analysis of healthy RM PBMC. G) Violin plots showing RNA expression levels of 4 genes of interest. H) Cytokine expression levels in CD101- and CD101+ memory CD4 after 3hr PMA/ionomycin stimulation of total PBMC from healthy individuals.

While CD101 has been investigated in chronic viral infection on CD8 T cell populations using the LCMV mouse model, its role on CD4 T cells has not been investigated in the rhesus macaque model and there have been no studies of CD101^+^ CD4 T cells in acute and chronic viral infection [34]. To understand the role of CD101 across CD4 T cell subsets in healthy animals, we performed flow cytometric analyses of cryopreserved peripheral blood mononuclear cells (PBMC), lymph node mononuclear cells (LN) and rectal mucosa biopsies (RB). While CD101 was not expressed on naïve CD4 T cells (CD28+ CD95-), it was moderately expressed on memory CD4 T cells (CD95+) and significantly enriched on memory CD4 Tregs (CD95+ CD25+ CD127- FoxP3+), with more than 60% of Tregs expressing CD101 in both blood and LN (Fig 1A, 1B and 1C) (S1 Fig). Despite the significant levels of CD101 expression on CD4 Tregs, they make up only a small fraction of the total CD101-expressing CD4 pool, as the overall contribution of CD101^+^ CD4 T cells largely comes from central and effector memory cells, due to their higher frequency of the total pool of CD4 T cells (Fig 1C). To further investigate the phenotype of CD101-expressing cells, we utilized Uniform Manifold Approximation and Projection (UMAP) of our flow cytometry analysis and focused on memory CD4 T cells. In combination with Phenograph analysis, this revealed distinct clusters of CD101^+^ memory CD4 T cells (Figs 1D and 1E and S2). In particular, CD101 was found highly expressed in clusters (2, 6, 17) also expressing markers of T regulatory cells, including CD25, FoxP3, CD39 and CTLA-4 (Fig 1D and 1E). An additional group of clusters (8, 10, 15, 23) confirmed the expression of CD101 in the central memory compartment, with co-expression of CCR7 and CD127 (Fig 1D and 1E).

Given the high expression of CD101 on CD4 Tregs, we performed single-cell RNA-seq (scRNA-seq) analysis on CD4 Tregs (CD95+ CD25+ CD127-) that were sorted into CD101^-^ and CD101^+^ populations. scRNA-seq analysis revealed clearly distinct transcriptional profiles of CD101^+^ Tregs as compared to CD101^-^ Tregs (Fig 1F and S1 File). Specifically, we found an expression profile suggesting CD101^+^ Tregs were more differentiated, with lower levels of *TCF7*, and showed higher potential for suppressive activity with lower levels of *SATB1* (a chromatin modulator that represses FoxP3 expression) and higher levels of *TNFRSF18* (which encodes for GITR), both of which have been strongly linked to the ability of Tregs to exert suppressive activity *in vivo* [35–37]. Additionally, CD101^+^ Tregs were highly enriched in expression of *LGALS3* (which encodes for Galectin-3), which has been shown to increase N-glycan branching on CD8 T cells, increasing the antigenic threshold for activation of these cells and contributing to long-term viral persistence in chronic LCMV model by limiting CD8 T cell antiviral functions [38].

To confirm that CD101^+^ cells represent a functionally distinct population, we performed PMA/ionomycin stimulation of PBMC from healthy individuals. After a 3hr stimulation, CD101^+^ CD4 T cells produced similar levels of traditional effector cytokines IL-2, IFNy, IL-17a and TNFa as compared to CD101- cells (Figs 1H and S3). However, CD101+ CD4 T cells produced significantly more LAP (Latency Associated Peptide; mean 11.7 fold-difference) than CD101- cells in response to stimulation. LAP is part of the latent TGF-β complex and is produced by Tregs that release TGF-β, suggesting that cells producing LAP are likely to be immunosuppressive [39–41]. Collectively, these observations suggest CD101-expressing cells represent a functionally distinct population of CD4 T cells that can play a key role in regulating inflammatory environments, such as those after a viral infection.

### CD101^+^ CD4 T cells are selectively depleted during SIV infection

**Fig 2:**
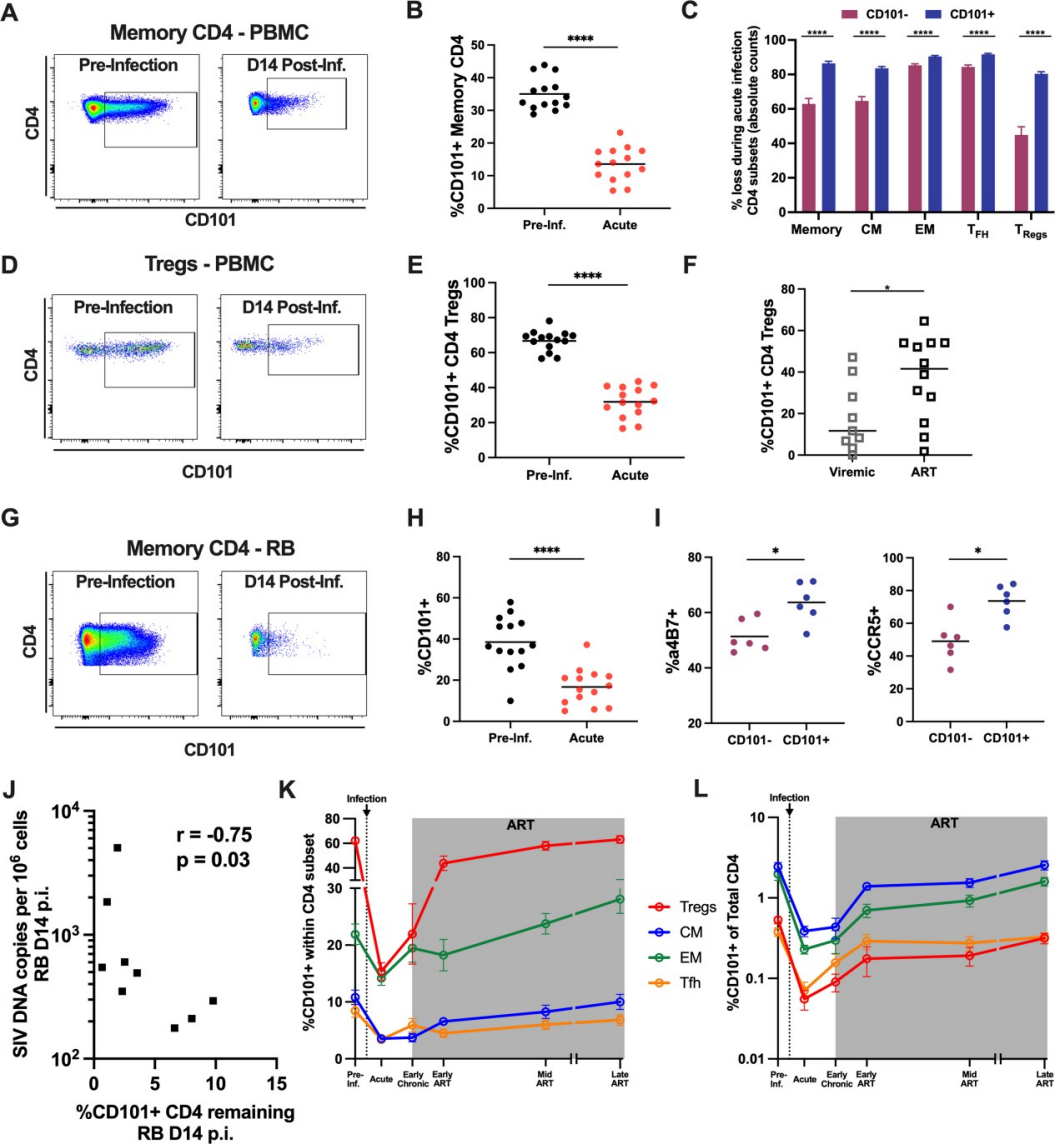
Dynamics of CD101-expressing CD4 T cells after SIV infection. A) Representative staining of CD101 on PBMC memory CD4 pre- and post-SIV infection. B) Expression levels of CD101 on PBMC memory CD4 pre- and post-SIV infection. C) Total loss of CD101^+^ and CD101^-^ cells within CD4 subsets calculated based on absolute counts in blood pre- and post-SIV infection (mean+SEM). D) Representative staining of CD101 on PBMC CD4 Tregs pre- and post-SIV infection. E) Expression levels of CD101 on PBMC CD4 Tregs pre- and post-SIV infection. F) Expression levels of CD101 on PBMC CD4 Tregs from HIV-infected individuals during active viremia and during suppressive antiretroviral therapy (ART). G) Representative staining of CD101 on rectal biopsy (RB) memory CD4 pre- and post-SIV infection. H) Expression levels of CD101 on RB memory CD4 pre- and post-SIV infection. I) Expression levels of α4β7 and CCR5 on RB memory CD4 CD101^-^ and CD101^+^ cells from healthy RM. J) Association between depletion of CD101+ CD4 T cells in rectal biopsies at day 14 p.i. (calculated from baseline to acute infection as % of live lymphocytes) and SIV DNA levels in rectal biopsies at day 14 p.i. (spearman correlation, n = 9). K) CD101 expression within PBMC CD4 subsets during longitudinal SIV infection (mean±SEM). L) Overall contribution of CD101^+^ cells to the overall CD4 pool from each PBMC CD4 subset during longitudinal SIV infection (mean±SEM). Lines designate mean values.

To determine the role of CD101-expressing CD4 T cells in SIV infection, we assessed the dynamics of CD101^+^ CD4 T cell populations in SIV_mac239_-infected RMs. CD101^+^ CD4 T cells are severely depleted during acute SIV infection (14 days post-infection), with a mean 2.6-fold reduction in the frequency of blood memory CD4 T cells expressing CD101 (Fig 2A and 2B). Absolute counts of CD101^+^ and CD101^-^ CD4 subsets in the periphery were evaluated to determine and compare the absolute loss of these populations during acute infection. CD101^+^ populations were significantly more depleted across all CD4 subsets as compared to CD101^-^ cells (Fig 2C). This preferential depletion was most severe for Tregs, as 80% of CD101^+^ Tregs were lost compared to only 44% of CD101^-^ Tregs, and for CM, with 84% of CD101^+^ cells lost compared to 64% of CD101^-^ (Fig 2C). Notably, CD4 Tregs shift from being a primarily CD101^+^ population (accounting for 66% of total Treg) to being majority CD101^-^ after SIV infection (only 32% of total Treg being CD101^+^), representing a shift during acute infection toward a population of Tregs with a reduced suppressive potential (Fig 2D and 2E). While depletion of CD101^+^ memory CD4+ T cells, or CD101^+^ Tregs, was not associated with viral load (copies HIV RNA/mL of plasma) or T cell activation at D14 p.i., there was a significant association between the loss of CD101^+^ cells and higher plasma viral load at D42 p.i. (Table 1). Additionally, lower levels of CD101^+^ cells at D42 p.i. was associated with increased plasma levels of the inflammatory cytokines IP-10 and MIP-1a (Table 1). These findings suggest a direct relationship between the depletion of CD101+ cells, an increased inflammatory environment and higher viral burden. Interestingly, increased CD8 T cell activation as assessed by levels of HLA-DR/CD38 was associated with better preservation of CD101+ CD4 T cell, suggestive of a potential for CD8 antiviral responses to limit the depletion of this population (Table 1).To expand this observation from SIV-infected RMs to humans, we assessed these populations in PBMCs from PWH and confirmed that CD101 expression on CD4 Tregs is significantly lower in individuals with active viremia compared to ART-suppressed individuals, despite equivalent overall frequencies of CD4 Tregs (Figs 2F and S4). To examine whether this depletion represented preferential susceptibility of CD101^+^ cells to HIV infection, we sorted CD101^-^ and CD101^+^ memory CD4 T cells from healthy human PBMC and performed an *in vitro* HIV infection. After 7 days in culture with ART, we harvested the cells and measured integrated HIV DNA, and found comparable levels of integrated HIV DNA between CD101^-^ and CD101^+^ cell cultures (S5A Fig).

**Table 1:** Correlations of CD101 dynamics with plasma viral load, immune activation and plasma cytokine levels. Analyses were done as Spearman correlations with r and p value displayed. All values are from the D14 p.i. or D42 p.i for 28 SIV-infected RMs. CD4 loss was calculated based on absolute counts of CD101+ CD4 pre- and post-infection. Statistically significant findings are highlighted in yellow (n = 28).

			Disease Progression (D14 p.i)			Disease Progression (D42 p.i)			Plasma Cytokine Levels (D42 p.i.)			
	Parameter	Spearman values	Plasma Viral Load	Memory CD4 HLA-DR+ CD38+	Memory CD8 HLA-DR+ CD38+	Plasma Viral Load	Memory CD4 HLA-DR+ CD38+	Memory CD8 HLA-DR+ CD38+	IL-6	IP-10	MIP-1a	TNF-a
**D14 p.i.**	**%CD101+ Memory CD4**	r	0.04	0.07	-0.01	-0.22	-0.02	0.04	0.05	-0.22	0.07	-0.16
		p	0.83	0.71	0.95	0.27	0.92	0.83	0.79	0.27	0.74	0.42
	**CD101+ Memory CD4 Counts**	r	0.20	0.24	0.22	-0.16	0.24	0.33	0.05	-0.19	0.25	-0.2
		p	0.30	0.23	0.26	0.41	0.22	0.09	0.79	0.34	0.21	0.32
	**CD101+ Memory CD4 Loss**	r	-0.05	-0.13	-0.13	0.01	0.03	0.14	0.29	0.02	-0.25	0
		p	0.79	0.52	0.51	0.95	0.1	0.48	0.89	0.92	0.21	0.99
	**%CD101+ on CD4 Treg**	r	-0.04	0.02	0.05	-0.08	-0.12	-0.09	0.03	-0.2	0.13	-0.19
		p	0.85	0.91	0.79	0.68	0.53	0.64	0.9	0.32	0.53	0.35
**D42 p.i.**	**%CD101+ Memory CD4**	r	-0.01	0.03	-0.1	-0.72	0.12	0.34	-0.05	-0.42	-0.38	-0.27
		p	0.97	0.9	0.61	0.0001	0.53	0.08	0.79	0.03	0.056	0.17
	**CD101+ Memory CD4 Counts**	r	0.03	0.21	0.13	-0.78	0.36	0.52	-0.09	-0.4	-0.27	-0.17
		p	0.87	0.29	0.51	0.0001	0.06	0.004	0.67	0.04	0.19	0.42
	**CD101+ Memory CD4 Loss**	r	0.03	-0.13	-0.002	0.79	-0.14	-0.41	0.25	0.23	0.43	0.02
		p	0.9	0.52	0.99	0.0001	0.48	0.03	0.23	0.27	0.03	0.92
	**%CD101+ on CD4 Treg**	r	-0.06	0.05	-0.04	-0.7	0.11	0.3	-0.22	-0.35	-0.39	-0.27
		p	0.75	0.81	0.84	0.0001	0.59	0.12	0.27	0.08	0.051	0.18

To understand if the preferential depletion of CD101^+^ cells was limited to peripheral blood or represented a migration of CD101^+^ CD4 to tissues, we evaluated these populations in rectal biopsy samples (RB) before and following SIV infection. Similar to PBMC, there was a marked, rapid (already present at d14 p.i.), depletion of CD101-expressing CD4 T cells in the gut after SIV infection (Fig 2G and 2H). This depletion was preferential, with 97% loss of CD101^+^ CD4 T cells compared to only 83% loss of CD101^-^ CD4 T cells (calculated from baseline to acute infection as % of live lymphocytes) (S6A Fig). Gut depletion of CD4 T cells is well described to be one of the main drivers of systemic inflammation and disease progression in viremic individuals during SIV/HIV infection, and the loss of highly suppressive CD101^+^ CD4 T cells could strongly contribute to this inflammation [42–44]. To investigate potential mechanisms for preferential depletion of CD101^+^ cells in the gut, we assessed levels of α4β7 and CCR5 on CD101^+^ and CD101^-^ memory CD4 T cells. These well-described surface receptors are known to drive T cell migration to the gut and to favor SIV/HIV cell entry. In uninfected macaques, CD101^+^ CD4 T cells in the gut express significantly more α4β7 and CCR5 than CD101^-^ cells (Fig 2I). This finding suggests CD101^+^ CD4 T cells have a high potential to migrate and reside in the gut tissues and is consistent with their propensity to be more depleted in the gut during acute SIV infection, especially given the role of α4β7-high cells as early targets for HIV/SIV infection and of CCR5 as a co-receptor for direct HIV/SIV infection [45]. To understand potential links between this depletion and viral burden in gut mucosa, we assessed SIV DNA and RNA levels in rectal biopsy samples from day 14 post-infection. We found a significant association between loss of CD101^+^ CD4 T cells (calculated from baseline to acute infection as % of live lymphocytes) and higher SIV DNA levels during acute infection (Fig 2J), while there was no association of SIV DNA/RNA levels with depletion of CD101^-^ CD4 T cells (S1 Table). This finding establishes a specific link between depletion of CD101^+^ cells in the mucosa and disease progression. Finally, we evaluated levels of circulating biomarkers of microbial translocation and mucosal barrier breakdown (sCD14, IFABp, zonulin) pre- and post-SIV infection. Notably, we found that lower levels of CD101^+^ cells in RB 14 days post-infection was significantly associated with a higher fold-change of zonulin from pre-infection to day 42 post-infection (S6B Fig) (S2 Table). Zonulin is a key regulator of intestinal permeability, with higher circulating levels of zonulin being indicative of intestinal epithelial damage, and has been previously shown to be predictive of HIV-related mortality [46].

To assess whether CD101 is being downregulated on CD4 T cells after infection rather than these cells being directly depleted, we evaluated CD101 surface levels after *in vitro* HIV infection of sorted CD101^-^ and CD101^+^ CD4 T cell cultures. Sorted CD101^+^ cells remained almost entirely CD101^+^ (median 92% CD101^+^) after 7 days in culture and remained clearly distinct from the CD101^-^ cultures, which remained CD101^-^ (S5B and S5C Fig). To confirm CD101 is not downregulated by T cell activation, we performed aCD3/CD28 stimulation of PBMC from healthy individuals. After 5 days of stimulation, CD101 levels on the total CD4 population had significantly increased (mean 2.5-fold increase) (S5D Fig).

Considering the potential inflammatory implications of a rapid and permanent depletion of highly suppressive Tregs, it is important to understand whether the depleted populations of CD101^+^ memory CD4 and Tregs are restored during antiretroviral therapy. To this aim, we quantified the frequency of blood CD4 T cell subsets expressing CD101 at an early (6 weeks), mid (24 weeks), and late (33–60 weeks) on ART time points. Within PBMC, the proportion of cells expressing CD101 within each CD4 subset was restored starting from early ART, and fully to pre-infection levels after long-term ART in RMs that initiated ART during the early chronic phase of infection (Fig 2K), thus suggesting direct viral infection or inflammation, both reduced by ART, as mechanisms for the loss of CD101-expressing cells. This restoration occurred very rapidly in the Treg compartment, suggesting the administration of ART prevents further depletion of CD101^+^ cells and allows a restoration of this suppressive population in response to the highly inflammatory environment. In fact, CD101^+^ cells begin to slowly recover prior to the initiation of ART, which is likely reflective of the tendency of Tregs to expand during the chronic phase of HIV/SIV infection and the emergence of SIV-specific CD8 T cells that are able to partially limit viremia during early chronic infection. The contribution of CD101^+^ cells within each subset to the overall pool of CD4 T cells was also fully restored to pre-infection levels (Fig 2L).

### During long-term ART, CD101^+^ cells display a phenotype consistent with quiescence and potential for reservoir persistence

**Fig 3:**
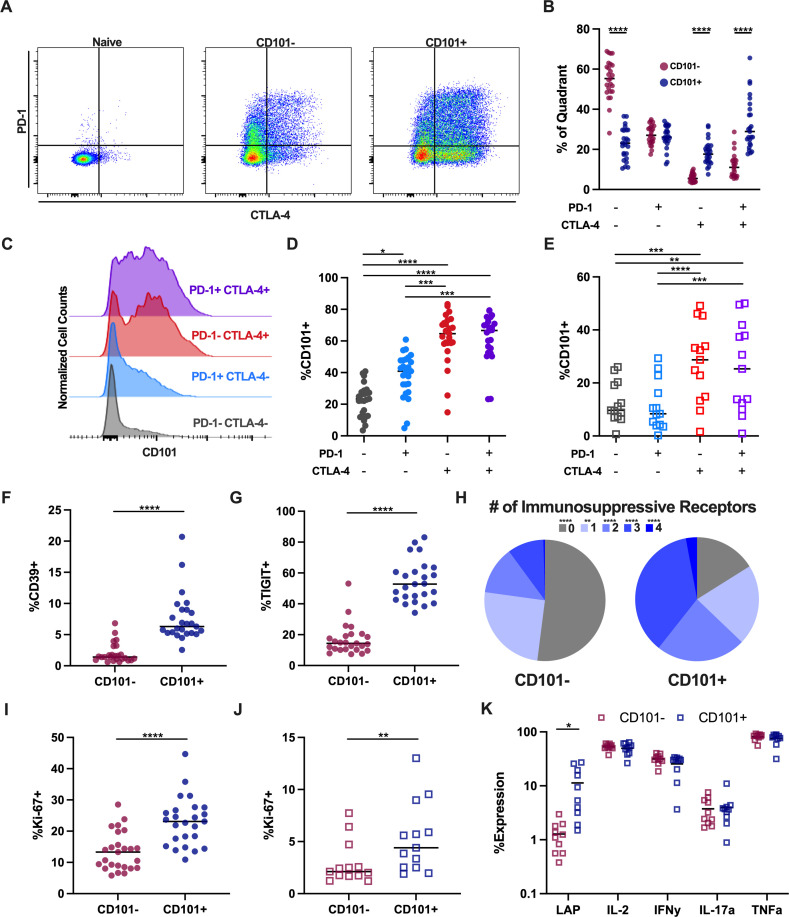
Phenotypic profile of CD101+ CD4 during long-term antiretroviral therapy. A) Representative staining of PD-1 and CTLA-4 within naïve, memory CD101^-^ and CD101^+^ CD4 T cells in lymph node (LN) from ART-suppressed SIV-infected RM. B) Quadrant distribution of PD-1 and CTLA-4 expression within CD101^-^ and CD101^+^ LN memory CD4 from ART-suppressed SIV-infected RM. C) Representative histogram of CD101 expression across PD-1 and CTLA-4 expression quadrants in LN memory CD4 from ART-suppressed SIV-infected RM. D) CD101 expression across PD-1 and CTLA-4 quadrants in LN memory CD4 from ART-suppressed SIV-infected RM. E) CD101 expression across PD-1 and CTLA-4 quadrants in PBMC memory CD4 from ART-suppressed HIV-infected individuals. F,G) Expression levels of CD39 (F) and TIGIT (G) within CD101^-^ and CD101^+^ LN memory CD4. H) Pie chart representing the frequency of cells expressing PD-1 and/or CTLA-4 and/or CD39 and/or TIGIT within CD101- and CD101^+^ LN memory CD4. I,J) Expression levels of Ki-67 within CD101^-^ and CD101^+^ memory CD4 from LN from ART-suppressed SIV-infected RM (I) and PBMC from ART-suppressed HIV-infected individuals (J). K) Cytokine expression levels in CD101- and CD101+ memory CD4 after 3hr PMA/ionomycin stimulation of total PBMC from ART-suppressed PLWH. Lines designate mean values.

We then investigated whether CD101^+^ cells may contribute to viral persistence during long-term ART following reconstitution. Expression of numerous inhibitory receptors on CD4 T cells have previously been described to potentially delineate subsets enriched for HIV/SIV provirus and contribute significantly to the persistent, latent reservoir [6,12,17,18,47]. Given the more differentiated state of CD101^+^ Tregs and associations of CD101 with inhibitory receptor expression on CD8 T cells during chronic LCMV infection, we hypothesized that CD101^+^ cells may be enriched for these markers during long-term ART [34]. We evaluated expression of PD-1 and CTLA-4 within the CD101^-^ and CD101^+^ memory CD4 populations in LN samples from SIV-infected RMs after >1 year of ART. While CD101^-^ cells primarily lie in the PD-1^-^ CTLA-4^-^ quadrant, CD101^+^ cells are significantly enriched in the PD-1^-^ CTLA-4^+^ and PD-1^+^ CTLA-4^+^ compartments (Fig 3A and 3B). Of note, we and others have previously reported that these populations of PD-1^-^ CTLA-4^+^ and PD-1^+^ CTLA-4^+^ cells harbor high levels of replication competent SIV DNA and include a high frequency of Tregs and Tfh, respectively [6,12,17]. Directly probing the expression of CD101 on the PD-1 and CTLA-4 quadrants of LN CD4 T cells revealed significantly elevated expression of CD101 in cells expressing any combination of PD-1 and CTLA-4 (Fig 3C and 3D). We confirmed similar results of CD101 expression on these cell populations in PBMC from PWH on ART (Fig 3E). We further investigated additional immunosuppressive receptors CD39 and TIGIT that are known to be expressed on functional Tregs and have been associated with the HIV reservoir [17,48–50]. CD101^+^ cells express significantly more CD39 and TIGIT as compared to CD101^-^ cells and Boolean gating on the 4 receptors of interest reveals a clear enrichment of immunosuppressive receptors on CD101^+^ cells, with 84% of CD101^+^ cells expressing at least one of these receptors compared to only 49% of CD101^-^ cells, and 63% of CD101^+^ cells expressing two or more receptors as compared to only 23% of CD101^-^ (Fig 3F, 3G and 3H). Additionally, we found that CD101^+^ cells express significantly higher levels of Ki-67 in both ART-suppressed SIV and HIV infection, suggestive of recent cell cycling of CD101^+^ cells (Fig 3I and 3J). This is notable considering the potential for cycling CD4 T cells to maintain the reservoir via clonal expansion [15,51]. Finally, to assess the functional capacity of CD101^+^ CD4 T cells to contribute to reservoir persistence, we performed PMA/ionomycin stimulation of PBMC from ART-suppressed PWH. Similar to the profile observed in cells from healthy individuals, CD101^+^ CD4 T cells expressed significantly more LAP (TGF-β) as compared to CD101^-^ cells, while producing similar levels of other effector cytokines (Fig 3K). This is particularly interesting, as TGF-β levels are known to be significantly elevated after HIV infection and remain elevated during suppressive ART and TGF-β has been demonstrated to play a role in potentiating T cell latency [52–56].

**Fig 4:**
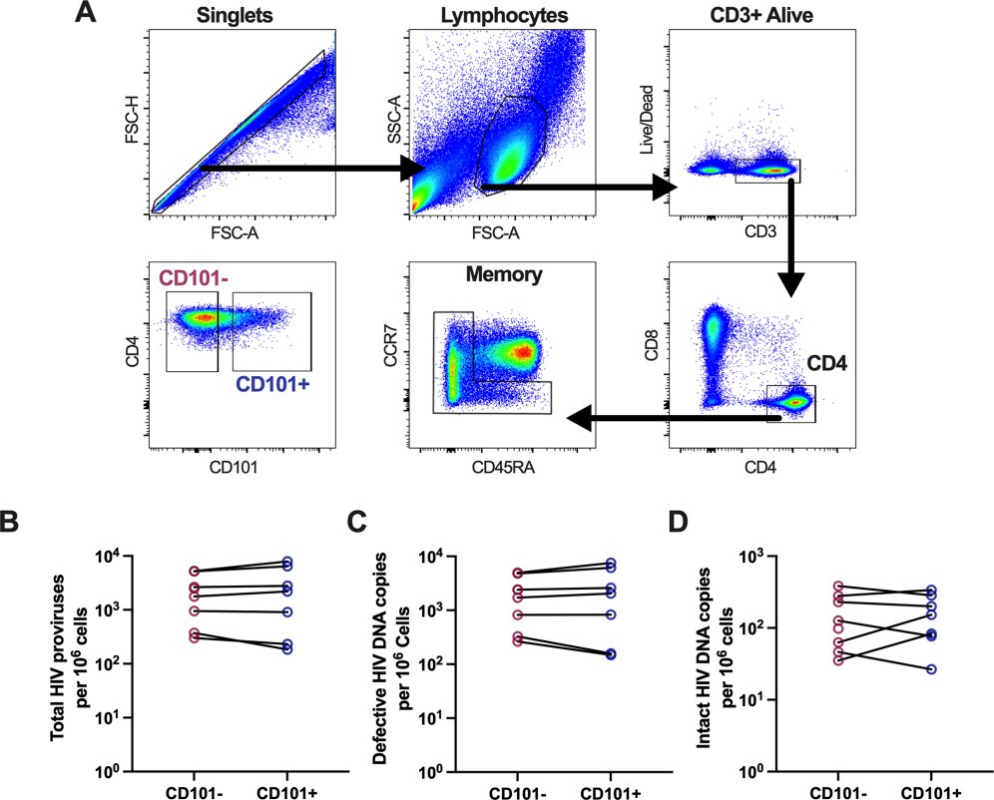
HIV DNA levels in sorted memory CD4 T cells. A) Sorting strategy for memory CD101^-^ and CD101^+^ memory CD4 T cells. B-D) Total HIV proviruses (B), defective HIV DNA copies (C) and intact HIV DNA copies (D) measured by IPDA in sorted CD101^-^ and CD101^+^ memory CD4 T cells from PBMC from ART-suppressed individuals.

To assess the potential contribution of CD101^+^ CD4 T cells to the latent HIV reservoir, we measured HIV DNA levels in sorted CD101^-^ and CD101^+^ memory CD4 T cells derived from PBMC from PWH on suppressive ART (Fig 4A). CD101^+^ CD4 harbored similar levels of total proviruses, defective HIV DNA, and intact HIV DNA as compared to CD101^-^ cells when assessed by the intact proviral DNA assay, suggesting that CD101^+^ cells are contributing to the overall pool of latently infected cells during long-term ART at levels comparable to the CD101^-^ counterpart (Fig 4B, 4C and 4D) [57]. Due to their high levels of intact DNA and a phenotype consistent with cell quiescence and survival, CD101^+^ CD4 cells have the potential to critically contribute to the long-term maintenance of the SIV/HIV reservoir.

## Discussion

Development of a functional cure for HIV continues to be the ultimate goal in HIV research, but these efforts are hampered by the difficult nature of HIV persistence. Many recent interventions have been aimed at reducing the size of the HIV reservoir, but the limited success to date suggests a need for further investigation into mechanisms maintaining the reservoir to help design targeted therapeutics. In this study, we characterize, for the first time, the phenotype and transcriptional profile of CD101^+^ CD4 T cells in NHPs, as well as their role in HIV/SIV pathogenesis and persistence. Our investigations reveal that CD101^+^ CD4 T cells are preferentially depleted during HIV/SIV infection in both blood and tissues, and this depletion is associated with increased viral burden in blood and gut and with elevated levels of inflammatory cytokines. While these cells are restored during long-term ART, they exhibit an immunosuppressive profile consistent with cells that critically contribute to the persistence of the latent viral reservoir.

In healthy RMs, we found that CD4 T cells expressing CD101 display a phenotypic and transcriptional profile suggestive of their role as highly functional Tregs, consistent with investigations of CD101^+^ CD4 T cells in other model systems and diseases. CD101^+^ Tregs express a transcriptional signature clearly differentiating them from CD101^-^ Tregs, with lower levels of *TCF7* and *SATB1*, indicative a more terminally differentiated Treg state, and higher levels of *TNFRSF18* and *LGALS3*, both of which have been shown to mark highly suppressive Treg populations [35–38]. Additionally, CD101^+^ CD4 T cells are more functionally suppressive, producing more LAP (TGF-β) in response to stimulation. Given the important role a highly suppressive CD4 population could play in both HIV pathogenesis during acute infection and HIV persistence during long-term ART, we performed the first longitudinal characterization of CD101^+^ CD4 T cells in both SIV and HIV infection. We found CD101^+^ CD4 T cells are preferentially depleted during acute SIV infection, with significantly larger reduction of CD101^+^ cells as compared to CD101^-^ cells across all CD4 subsets. Importantly, this depletion was associated with higher viral load and increased levels of inflammatory cytokines, suggesting that depletion of CD101^+^ cells is directly linked to a more inflammatory environment. The depletion of CD101^+^ CD4 T cells was most pronounced within the Treg compartment. While the frequency of Tregs has been shown to increase during chronic HIV infection, few studies have been able to address the role and dynamics of CD4 Tregs during acute infection, and conclusions from these studies vary on whether Tregs are increased or decreased during acute infection [58–64]. Our findings that CD101^+^ immunosuppressive Tregs are depleted during acute infection suggest an additional mechanism for increased immune activation during primary HIV infection. Additionally, the confirmation of persistently lower levels of CD101 on Tregs, despite equivalent frequencies of Tregs, in viremic PWH as compared to ART-suppressed individuals implies the persistent inflammatory environment in this setting may be partially due to the lack of highly functional Tregs. The depletion of immunosuppressive CD101^+^ cells may be most impactful in the gut mucosa, where loss of CD4 T cells is known to drive systemic mucosal barrier breakdown and systemic inflammation [43,65–69]. We show that CD101^+^ cells express higher levels of CCR5 and α4β7 than CD101^-^ cells, providing rationale for their homing and selective depletion in gut tissue, as both are known to mark cells preferentially infected with HIV/SIV [45,70,71]. Furthermore, depletion of α4β7^+^ CD4 T cells during acute HIV/SIV infection is associated with increases in microbial translocation, a key mechanism for SIV/HIV pathogenesis, suggestive of more extensive gut damage [45]. Indeed, we found that decreased levels of CD101^+^ cells in the gut during acute infection was associated with higher fold-change of zonulin, a marker of intestinal epithelial integrity, during early chronic infection. Additionally, depletion of these cells was associated with increased viral burden (SIV DNA) in the mucosa during acute infection, suggesting that loss of CD101^+^ CD4 T cells in the gut mucosa may play an important role in increased activation, infection and mucosal breakdown as HIV/SIV infection progresses.

While the preferential loss of highly immunosuppressive CD101^+^ CD4 T cells is likely contributing to pathogenesis during primary HIV/SIV infection, cells of this nature also likely contribute to reservoir persistence during long-term ART. Investigations over recent years have yielded strong evidence that expression of inhibitory receptors, including PD-1, CTLA-4 and TIGIT, contribute to HIV persistence [6,12,17,18]. As suggested by the differentiation state of these cells and previous reports of CD101 expression on CD8 in viral infection, we demonstrate that CD101^+^ CD4 T cells are highly enriched for expression of immunosuppressive receptors in lymphoid tissue during ART. Interestingly, there is a significant enrichment for CD101^+^ CD4 T cells within CTLA-4^+^ PD-1^-^ cells, which our group has previously shown to be an important contributor to viral persistence within the lymphoid tissue [12]. Additionally, CD101^+^ cells express higher levels of Ki-67, indicative of their ability to proliferate and persist over time on ART and potentially maintain the reservoir through clonal expansion. While CD101^+^ cells did not show enrichment for HIV DNA as compared to CD101^-^ cells in blood, they harbor significant levels of intact HIV DNA and we believe their contribution to viral persistence may still be critical. First, expression of inhibitory receptors is one way in which HIV-infected CD4 T cells maintain latency, and blockade of these receptors can potentiate latency reversal [47,72,73]. Second, CD101^+^ CD4 T cells, due to their highly functional regulatory potential, likely create an immunosuppressive environment that prevents HIV expression and CD8-mediated clearance of infected cells. The enriched production of LAP (TGFβ) by CD101^+^ CD4 T cells compared to CD101- cells in samples from PWH is likely to promote latency of these cells, as recent work has highlighted the important role of TGFβ in promoting reservoir persistence [53–56,74,75]. Additionally, the observation that CD101^+^ Tregs have higher expression of *LGALS3* is particularly interesting in the context of previous findings that galectin-3 participates in N-glycan branching on CD8 T cells during chronic infection that increases the antigenic threshold for CD8 T cell activation and function [38]. This suggests that CD101^+^ CD4 T cells during long-term ART could inhibit CD8-mediated clearance of HIV-infected cells in reservoir clearance attempts utilizing a shock and kill strategy.

It is difficult to assess direct cell death *in vivo*, and therefore we cannot say for certain that CD101 cells are preferentially depleted due to increased infection and cell death. However, it is unlikely that the loss of CD101^+^ cells is due to a redistribution of these cells throughout tissues, as we assessed these populations in both blood and mucosal tissue and found depletion in both. Furthermore, it seems unlikely CD101 is downregulated on cells given the terminally differentiated status of these cells, the upregulation of CD101 on TCR-stimulated CD4 T cells, and the lack of downregulation observed in sorted CD101^+^ cells after 7 days in our *in vitro* HIV infection system. Additionally, although our *in vitro* infection model does not show increased infection levels of CD101^+^ CD4 T cells, this model may not fully replicate the phenotypic environment of these cells during *in vivo* infection, particularly given the role of CD101^+^ cells in the gut. Unfortunately, given the very low levels of CD101^+^ CD4 T cells after infection, we are unable to sort these cells and directly assess whether they harbor more virus at this time. The idea that CD101 expression is associated with preferential infection and depletion is additionally supported by two independent studies in which genetic variants in the *CD101* locus are associated with altered immune activation and increased risk of sexually acquired HIV infection [76, 77]. While we did not find significant enrichment of HIV DNA in CD101^+^ cells from PBMC, we were unable to assess either LN and rectal biopsy samples for these measures, where we believe the impact of both preferential depletion of CD101^+^ CD4 T cells and the immunosuppressive environment created by them on ART may be most significant.

Continued exploration of markers and mechanisms of cells contributing to HIV pathogenesis and persistence is vital to designing more effective interventions focused on HIV cure. Herein, we report the first comprehensive assessment of the role of CD101^+^ CD4 T cells in HIV/SIV infection, in which we define CD101^+^ cells as a marker of highly suppressive CD4 Tregs, a population that is preferentially and severely depleted during acute infection and restored during long-term ART, with the potential to contribute to and promote the persistence of the HIV reservoir. Additional studies assessing how modulation of CD101 expression on CD4 T cells can impact viral pathogenesis and reservoir persistence may reveal additional strategies for HIV therapeutics.

## Materials and methods

### Ethics statement

All animal experimentation was conducted following guidelines set forth by the Animal Welfare Act and by the NIH’s Guide for the Care and Use of Laboratory Animals, 8th edition. All studies were reviewed and approved by Emory’s Institutional Animal Care and Use Committee (IACUC; permit numbers 201700655, 201800047, 201700665) and animal care facilities at Yerkes National Primate Research Center are accredited by the U.S. Department of Agriculture (USDA) and the Association for Assessment and Accreditation of Laboratory Animal Care (AAALAC) International. Proper steps were taken to minimize animal suffering and all procedures were conducted under anesthesia with follow-up pain management as needed. Individuals with HIV infection were consented for a study approved by the Emory University Institutional Review Board and written consent for research was obtained from the individuals.

### Animals, SIV infection, antiretroviral therapy and sample collection

53 Indian rhesus macaques (RMs), housed at the Yerkes National Primate Research Center, were included in this study. All RMs were screened to ensure Mamu-B*08- and Mamu-B*17- status. Animals were infected intravenously with 300 TCID50 SIVmac239. Animals initiated a daily subcutaneous antiretroviral therapy regimen of FTC (40 mg/kg), TDF (5.1 mg/kg) and DTG (2.5 mg/kg) during the early chronic phase of infection (week 6–8 post-infection) and were maintained on ART up to 66 weeks post-infection (S3 Table). Peripheral blood, lymph node and rectal biopsy sample collections were conducted at critical timepoints during the study and processed as previously described [12]. Infection timepoints were defined as follows: acute (days 14–18 post-infection), early chronic (days 42–56 post-infection), early ART (6 weeks post-ART initiation), mid ART (24 weeks post-ART initiation), late ART (33–60 weeks post-ART initiation).

### Samples from participants with HIV infection

For analysis of depletion of CD101-expressing CD4 T cells during viremia, PBMC were obtained prior to initiation of antiretroviral therapy in individuals with a confirmed HIV diagnosis. For analysis of CD101 expression during long-term antiretroviral therapy, PBMC were obtained from individuals with undetectable viremia for at least 1 year prior to sample collection. Individuals were on a variety of combination ART regimens containing a protease inhibitor, integrase inhibitor and/or nucleoside reverse-transcriptase inhibitors. Cryopreserved cells were used for all analyses.

### Plasma viral load

Levels of SIV RNA in plasma were measured by RT-qPCR as previously described (limit of detection 60 copies/mL) [78].

### scRNA-seq analysis

Single cells were sorted directly into 96 well plates with lysis buffer. Single cell lysates were then converted to cDNA following capture with Agencourt RNA Clean beads using the SmartSeq2 protocol as previously described [79]. The cDNA was amplified using 20–24 PCR enrichment cycles prior to quantification and dual-index barcoding with the Illumina Nextera XT kit. The libraries were enriched with 12 cycles of PCR, then combined in equal volumes prior to final bead cleanup and sequencing. All libraries were sequenced on an Illumina HiSeq 3500 by either single-end 150 bp reads or short paired-end reads. Alignment was performed using STAR version 2.5.2b and transcripts were annotated using MacaM Rhesus genome assembly and annotation (v7.8.2: https://www.unmc.edu/rhesusgenechip/index.htm#NewRhesusGenome) [80]. Transcript abundance estimates were calculated internal to the STAR aligner using the algorithm of htseq-count [81].

### Flow cytometry

Flow cytometric analyses for this study were performed on peripheral blood, LN and gut-derived cells according to previously optimized and standardized procedures with anti-human antibodies with confirmed cross-reactivity with RMs. For T cell phenotyping analyses, the following antibodies were used at pre-optimized staining concentrations: anti-Ki-67-Alexa700 (clone B56), anti-CD3-BUV395 (clone SP34-2), anti-CD8-BUV496 (clone RPA-T8), anti-CD45-BUV563 (clone D058-1283),anti-CD28-BUV737 (clone CD28.2), anti-CD45RA-BUV737 (clone HI100), anti-CTLA-4-BV421 (clone BNI3), anti-Ki-67-BV480 (clone B56), anti-CD27-BV605 (clone L128), anti-CCR5-APC (clone 3A9), anti-CCR7-BB700 (clone 3D12), Fixable Viability Stain 700 (all from BD Biosciences); anti-FoxP3-AF647 (clone 150D), anti-CD4-APC/Cy7 (clone OKT4), anti-CD95-BV605 (clone DX2), anti-HLA-DR-BV650 (clone L243), anti-CD25-BV711 (clone BC96), anti-CD39-BV711 (clone A1), anti-PD-1-BV786 (clone EH12.2H7), anti-CD39-PE/Dazzle594 (clone A1), anti-CD4-PE/Cy5 (clone OKT4), anti-CD101-PE/Cy7 (clone BB27) (all from Biolegend); anti-CXCR5-PE (clone MU5UBEE), anti-CD127-Pe/Cy5 (clone ebioRDR5), anti-TIGIT-PerCP-eF710 (clone MBSA43), LIVE/DEAD fixable aqua (all from ThermoFisher); anti-CD38-FITC (clone AT-1) (StemCell); anti-α4β7-PE (clone A4B7R1) (NHP reagent resource). To detect intracellular expression of FoxP3, cells were fixed and permeabilized with FoxP3 Fix/Perm solution (Tonbo) and subsequently stained for intracellular markers of interest. Acquisition of stained cells was performed on a minimum of 100,000 live CD3+ T cells for LN and PBMC samples on an LSRFortessa or FACSymphony (BD biosciences) driven by FACSDiva software and analyzed using FlowJo software (version 10.8, Treestar). FCS files were imported into FlowJo, compensated electronically, gated on memory CD4 T cells (CD3+ CD4+ CD8- CD95+) and equivalent numbers of cells were input into UMAP analysis (Uniform Manifold Approximation and Projection for Dimension Reduction) was for unbiased evaluation of the distribution of the key markers. Projection of the density of cells expressing markers of interest were visualized/plotted on a 2-dimensional UMAP (https://arxiv.org/abs/1802.03426, https://github.com/lmcinnes/umap). We used the Phenograph clustering approach (https://github.com/jacoblevine/PhenoGraph).

### Flow cytometry cell sorting

In order to perform scRNA-seq on sorted CD101+ and CD101- Tregs, cryopreserved PBMC were thawed and stained with the following antibodies: anti-CD95-BV421 (clone DX2), anti-CD28-PE/CF594 (clone 28.2) (from BD Biosciences); anti-CD4-BV650 (clone OKT4), anti-CD101-PE/Cy7 (clone BB27) (from Biolegend); anti-CD8-FITC (clone MHCD08014), anti-CXCR5-PE (clone MU5UBEE), anti-CD127-PE/Cy5 (clone ebioRDR5), LIVE/DEAD fixable aqua (from ThermoFisher). Cells were gated as live, CD3+, CD4+, CD8- and were then sorted as Naïve (CD28+ CD95-), CD101- Tregs (CD95+, CXCR5-, CD127-, CD25+, CD101-) and CD101+ Tregs (CD95+, CXCR5-, CD127-, CD25+, CD101+).

For HIV DNA measurements in CD101+ and CD101- CD4 T cells, cryopreserved PBMC were thawed and stained with the following antibodies: anti-CCR7-BB700 (clone 3D12), anti-CD45RA-APC (clone HI100), anti-CD3-APC-Cy7 (clone SP34-2), anti-CD8-FITC (clone RPA-T8) (from BD biosciences); anti-CD4-APC/Cy7 (clone OKT4), anti-CD101-PE/Cy7 (clone BB27) (from Biolegend). Cells were sorted as memory CD101^+^ or CD101^-^ CD4 (gating strategy shown in Fig 4A) and DNA was extracted using a QIAmp DNA minikit (Qiagen) and intact proviral DNA was measured as previously published, with sample processing and IPDA analysis performed by Accelevir Diagnostics in a blinded fashion [57].

### In vitro infection

*In vitro* infection of PBMC was performed as previously described [56]. Briefly, PBMC were isolated from buffy coats from HIV-negative healthy donors and enriched for memory CD4 T cells by negative selection using the EasySep human CD4 memory T cell enrichment kit (StemCell). Cells were rested overnight and then sorted as CD101^+^ or CD101^-^ using a FACS Aria II system (BD Biosciences). After another overnight rest, cells were spinoculated with 89.6, a clinical isolate of HIV, at 100ng/mL p24/million cells. Viral supernatant was removed and infected cells were cultured in RPMI supplemented with 30 U/mL IL-2 (R&D Systems) and 5uM saquinavir (NIH AIDS Reagent Program) to prevent viral spread and preintegration latency. On day 4, cells were directed into latency with addition of 40ng/mL recombinant human IL-7 (R&D Systems), 20 ng/mL recombinant human TGF-B1 (PeproTech) and an ART cocktail of 5uM saquinavir, 100nM efavirenz and 200nM raltegravir (NIH AIDS Reagent Program). Cells were harvested on day 7 for flow cytometric analysis and measurements of integrated HIV DNA levels. The following antibodies were used for analysis: anti-CD3-BUV395 (clone SP34-2), anti-CD8-BUV496 (clone RPA-T8), anti-CD45RA-BUV737 (clone HI100), anti-CD27-BV605 (clone L128), anti-CCR7-BB700 (clone 3D12), anti-CD45RA-APC (clone HI100), anti-CD3-BV421 (clone SP34-2), anti-CD8-PE/CF594 (clone RPA-T8), (from BD biosciences); anti-CD4-APC/Cy7 (clone OKT4), anti-CD101-PE/Cy7 (clone BB27) (from Biolegend). Acquisition of stained cells was performed on an LSRFortessa or FACSymphony (BD biosciences) driven by FACSDiva software and analyzed using FlowJo software (version 10.8, Treestar). Measurements of integrated HIV DNA on cells from day 7 was performed as previously described [82].

### Measurement of intestinal epithelial damage biomarkers

Levels of circulating biomarkers of mucosal damage in plasma were measured by enzyme-linked immunosorbent assay (ELISA) using a commercially available kit as per the manufacturer’s instructions (sCD14: R&D Systems, Cat #DC140l; IFABP: My Biosource, Cat#MBS740424; zonulin: Alpco, Cat #30-ZONSHU-E01).

### In vitro stimulations

*In vitro* stimulations were performed on total PBMC from both healthy donors and ART-suppressed PLWH. For PMA/ionomycin stimulations, cells were stimulated at 37°C with 80 ng/mL PMA and 500 ng/mL ionomycin in the presence of 10ug/mL of BFA and 0.7uL of GolgiStop (BD biosciences). Cells were harvested and stained at 0hr and 3hr post-stimulation, with the 0hr timepoint serving as a negative control. For aCD3/aCD28 stimulations, cells were cultured in RPMI supplemented with 10% heat-inactivated FBS, 100U/mL penicillin, 100ug/mL streptomycin and 20U/mL recombinant human IL-2 (Gemini Bio-products), 5ug/mL anti-CD28-BUV737 (clone CD28.2, BD biosciences), 5ug/mL anti-CD2-purified (clone RPA-2.10, Biolegend), and 5ug/mL anti-CD3-purified (clone SP34-2, BD Biosciences). Fresh IL-2 at 20U/mL was supplemented every 2 days and cells were harvested and stained at days 0, 3 and 5 post-stimulation. For downstream flow cytometry analysis, the following antibodies were used: anti-CD4-APC/Cy7 (clone OKT4), anti-PD-1-BV786 (clone EH12.2H7), anti-CD101-PE/Cy7 (clone BB27), anti-IFNg-PE/Dazzle594 (clone B27), anti-TNFa-APC (clone MAb11), anti-IL-2-BV650 (clone MQ1-17H12), anti-LAP-BV421 (clone TW4-2F8) (From Biolegend); anti-Ki-67-Alexa700 (clone B56), anti-CD3-BUV395 (clone SP34-2), anti-CD8-BUV496 (clone RPA-T8), anti-CD45RA-BUV737 (clone HI100)), anti-CD27-BV605 (clone L128), anti-CCR7-BB700 (clone 3D12) (from BD biosciences); anti-IL-17a-AF488 (clone eBio64DEC17) from eBioscience; LIVE/DEAD fixable aqua from ThermoFisher.

### Plasma cytokine levels

Plasma cytokines were measured using the MSD platform as per manufacturer’s instruction with NHP-specific U-PLEX for IL-6 (K156TXK), IP-10 (K156UFK), MIP-1a (K156UJK), TNF-a (K156UCK) from plasma samples from day 42 post-infection. Values below limit of detection were imputed at half of the limit of detection.

### SIV DNA/RNA measurements

Levels of SIV DNA and RNA in rectal mucosa were measured as previously described [83, 84]. Briefly, rectal biopsy tissue was homogenized, RNA and DNA were extracted and SIV DNA and RNA levels were measured by qRT-PCR and normalized to cell counts using concurrent rhesus albumin measurements.

### Statistical analyses

Analyses were performed using R studio or GraphPad Prism 9. *P* values ≤ 0.05 were considered statistically significant with the following definitions: *P < 0.05, **P < 0.01, ***P < 0.001, ****P < 0.0001. Differences in unmatched data were evaluated using Mann-Whitney U test and matched data were evaluated using Wilcoxon matched-pairs signed rank test. For samples with multiple comparisons, values were adjusted for multiple comparisons using Dunn’s test, false-discovery rate or Sidak correction. Longitudinal analyses were evaluated using mixed-effects model with Tukey correction. Correlations were calculated using nonparametric Spearman analysis.

## Supporting information

S1 Table Association of CD101 gut depletion and mucosal viral burden. Analyses were done as Spearman correlations with r and p value displayed. %Remaining (amount of depletion) was calculated from baseline to d14 p.i. as % of live lymphocytes. Statistically significant findings are highlighted in yellow (n = 9).
(PDF)

S2 Table Intestinal epithelial damage biomarker correlations. Analyses were done as Spearman correlations with r and p value displayed. Fold-change of biomarkers was assessed as change from pre-infection baseline to either d14 or d42 post-infection. Statistically significant findings are highlighted in yellow (n = 13).
(PDF)

S3 Table Animal characteristics. (PDF)

S1 Fig CD101 in healthy RM. A) Representative gating strategy for CD4 subsets within LN samples from healthy rhesus macaques. B) CD101 expression on CD4 subsets in rectal biopsy samples from healthy rhesus macaques. Lines designate means.
(TIFF)

S2 Fig UMAP with phenograph cluster locations. (TIFF)

S3 Fig Representative gating for cytokine expression after stimulation. Representative gates for cytokine levels within memory CD4 at 0hr post-stimulation and within CD101- and CD101+ memory CD4 at 3hr post-stimulation with PMA/ionomycin.
(TIFF)

S4 Fig Frequency of CD4 Tregs in HIV-infected individuals. The frequency of Tregs (CD25+ CD127- FoxP3+) within the memory pool of CD4 Tregs evaluated in PBMC from viremic and ART-suppressed individuals. Lines designate means.
(TIFF)

S5 Fig *In vitro* infection of sorted CD4 T cells and exclusion of CD101 downregulation. A) Levels of integrated HIV DNA from CD101- or CD101-positive cell cultures 7 days after *in vitro* infection of sorted cells. B) Overlayed flow plot of expression of CD101 on CD4 T cells from CD101- (maroon) and CD101+ (blue) cultures 7 days after *in vitro* infection of sorted cells. C) Gated expression levels of CD101 on CD4 T cells from CD101- and CD101+ cultures. D) CD101 expression levels on total CD4 T cells after aCD3/aCD28 stimulation of PBMC from healthy individuals.
(TIFF)

S6 Fig Preferential depletion of CD101+ CD4 T cells in the gut. A) Loss of CD101- and CD101+ CD4 T cells in the gut during acute infection, calculated as %loss from baseline using the frequency of CD101- and CD101+ cells of total live lymphocytes. B) Association between levels of CD101-expressing CD4 T cells in the gut during acute infection and fold change of circulating zonulin in plasma at day 42 p.i. compared with pre-infection (spearman correlation with linear regression and 95% confidence interval).
(TIFF)

S1 File All statistically significant differentially expressed genes between CD101- and CD101+ Tregs by scRNAseq. (XLSX)
