# Supplementary material for: The role of CD101-expressing CD4 T cells in HIV/SIV pathogenesis and persistence
Source: PLoS Pathog. 2022 Jul 22;18(7):e1010723. doi: 10.1371/journal.ppat.1010723 (PMC9348691; doi:10.1371/journal.ppat.1010723)
Supplement: S1 Table — Analyses were done as Spearman correlations with r and p value displayed. %Remaining (amount of depletion) was calculated from baseline to d14 p.i. as % of live lymphocytes. Statistically significant findings are highlighted in yellow (n = 9). (PDF) [file ppat.1010723.s001.pdf]

		SIV DNA RB D14 p.i.	SIV RNA RB D14 p.i.
%CD101+ Memory CD4 RB D14 p.i.	r	-0.13	0.32
	p	0.74	0.41
%Depletion of CD101+ CD4 RB D14 p.i.	r	-0.75	0.55
	p	0.03	0.13
%Depletion of CD101- CD4 RB D14 p.i.	r	0.52	0.53
	p	0.16	0.15
