# Supplementary material for: The role of CD101-expressing CD4 T cells in HIV/SIV pathogenesis and persistence
Source: PLoS Pathog. 2022 Jul 22;18(7):e1010723. doi: 10.1371/journal.ppat.1010723 (PMC9348691; doi:10.1371/journal.ppat.1010723)
Supplement: S2 Table — Analyses were done as Spearman correlations with r and p value displayed. Fold-change of biomarkers was assessed as change from pre-infection baseline to either d14 or d42 post-infection. Statistically significant findings are highlighted in yellow (n = 13). (PDF) [file ppat.1010723.s002.pdf]

		D14 p.i.			D42 p.i.		
		FC Zonulin	FC sCD14	FC IFABp	FC Zonulin	FC sCD14	FC IFABp
%CD101+ Memory CD4 RB D14 p.i.	r	-0.2527	0.1758	0.2582	-0.5874	0.2198	0.3626
	p	0.4043	0.5659	0.3939	0.0489	0.4703	0.224
%CD101+ Memory CD4 RB D42 p.i.	r	-0.5385	0.01099	-0.3132	-0.5804	0.3297	0.2747
	p	0.0611	0.9782	0.2975	0.0521	0.2715	0.3633
