# Supplementary material for: The role of CD101-expressing CD4 T cells in HIV/SIV pathogenesis and persistence
Source: PLoS Pathog. 2022 Jul 22;18(7):e1010723. doi: 10.1371/journal.ppat.1010723 (PMC9348691; doi:10.1371/journal.ppat.1010723)
Supplement: S3 Table — (PDF) [file ppat.1010723.s003.pdf]

	Virus	Sex	Age at Infection (Months)	Mamu A*01	Acute Plasma Viral Load (copies/mL)	Pre-ART Plasma Viral Load (copies/mL)	ART Initiation (week post-infection)	Time on ART (weeks)	Figures
RFl17	SIVmac239	M	44	+	1.23E+07	8.87E+05	6	60	1 D-E, 2 A-E, 3A-D, 3F-I
RYk17	SIVmac239	M	45	+	7.92E+06	8.02E+03	6	60	1 D-E, 2 A-E, 3A-D, 3F-I
RVz16	SIVmac239	M	47	+	6.02E+06	4.04E+05	6	60	1 D-E, 2 A-E, 3A-D, 3F-I
RYm17	SIVmac239	M	44	+	1.16E+07	1.28E+06	6	60	1 D-E, 2 A-E, 3A-D, 3F-I
RHm17	SIVmac239	M	44	-	9.68E+06	9.10E+06	6	60	1 D-E, 2 A-E, 3A-D, 3F-I
RNy16	SIVmac239	M	48	+	1.45E+07	2.33E+05	6	60	1 D-E, 2 A-E, 3A-D, 3F-I
RTd17	SIVmac239	M	46	+	2.16E+07	1.33E+06	6	60	1 D-E, 2 A-E, 3A-D, 3F-I
34897	SIVmac239	M	45	-	8.23E+06	1.15E+06	6	60	1 D-E, 2 A-E, 3A-D, 3F-I
RRh17	SIVmac239	M	45	-	8.98E+06	7.97E+04	6	60	1 D-E, 2 A-E, 3A-D, 3F-I
RFn17	SIVmac239	M	44	-	1.66E+07	4.11E+06	6	60	1 D-E, 2 A-E, 3A-D, 3F-I
RVm17	SIVmac239	M	44	-	5.81E+06	2.68E+05	6	60	1 D-E, 2 A-E, 3A-D, 3F-I
RBe17	SIVmac239	M	46	-	1.13E+07	6.92E+05	6	60	1 D-E, 2 A-E, 3A-D, 3F-I
RMl17	SIVmac239	M	44	+	1.90E+07	4.67E+06	6	60	1 D-E, 2 A-E, 3A-D, 3F-I
RIb17	SIVmac239	M	47	-	4.26E+07	1.39E+07	6	60	1 D-E, 2 A-E, 3A-D, 3F-I
34918	SIVmac239	F	48	-	2.00E+07	1.25E+06	6	60	2G-H, 2J, 3A-D, 3F-I
RBf17	SIVmac239	M	50	-	2.88E+07	5.32E+06	6	60	2G-H, 2J, 3A-D, 3F-I
RBv17	SIVmac239	M	38	-	2.88E+07	2.28E+05	6	60	2G-H, 3A-D, 3F-I
REu17	SIVmac239	F	38	-	1.70E+07	2.32E+05	6	60	2G-H, 2J, 3A-D, 3F-I
RKq17	SIVmac239	M	39	-	3.04E+07	1.11E+06	6	60	2G-H, 3A-D, 3F-I
RVc17	SIVmac239	M	50	-	2.90E+07	6.11E+05	6	60	2G-H, 2J, 3A-D, 3F-I
RZg17	SIVmac239	M	49	-	1.92E+07	2.06E+05	6	60	2G-H, 3A-D, 3F-I
34920	SIVmac239	F	48	-	6.74E+07	4.68E+06	6	60	2G-H, 2J, 3A-D, 3F-I
34930	SIVmac239	M	48	-	3.50E+07	2.36E+06	6	60	2G-H, 3A-D, 3F-I
REq17	SIVmac239	M	39	-	2.92E+07	2.79E+05	6	60	2G-H, 2J, 3A-D, 3F-I
RGr17	SIVmac239	M	39	-	1.10E+07	1.05E+07	6	60	2G-H, 3A-D, 3F-I
RVi17	SIVmac239	M	49	+	2.42E+07	2.50E+07	6	60	2G-H, 2J, 3A-D, 3F-I
RWs17	SIVmac239	M	38	-	1.07E+07	5.60E+05	6	60	2G-H, 2J, 3A-D, 3F-I
RZp17	SIVmac239	M	39	-	4.73E+06	5.10E+04	6	60	2G-H, 2J, 3A-D, 3F-I
RAg16	SIVmac239	M	41	-	6.70E+07	3.60E+07	8	44	2K-L
RGt16	SIVmac239	M	28	-	3.60E+07	2.60E+06	8	50	2K-L
RHk16	SIVmac239	M	30	-	1.50E+07	3.20E+04	8	33	2K-L
RIs16	SIVmac239	M	29	+	2.00E+07	1.70E+06	8	34	2K-L
RLw16	SIVmac239	M	29	-	1.60E+07	1.20E+07	8	41	2K-L
RNi16	SIVmac239	M	32	-	5.80E+07	3.10E+07	8	44	2K-L
RQj16	SIVmac239	M	31	-	2.20E+08	2.40E+07	8	46	2K-L
RWn16	SIVmac239	M	30	+	3.20E+07	3.50E+06	8	37	2K-L
RIa17	Uninfected			N/A	N/A		N/A	N/A	2I
RSd17	Uninfected			N/A	N/A		N/A	N/A	2I
RSk17	Uninfected			N/A	N/A		N/A	N/A	2I
RTb17	Uninfected			N/A	N/A		N/A	N/A	2I
RZe17	Uninfected			N/A	N/A		N/A	N/A	2I
RZn17	Uninfected			N/A	N/A		N/A	N/A	2I
93-11R	Uninfected			N/A	N/A		N/A	N/A	1A-C
97-11R	Uninfected			N/A	N/A		N/A	N/A	1A-C
103-11R	Uninfected			N/A	N/A		N/A	N/A	1A-C
112-11R	Uninfected			N/A	N/A		N/A	N/A	1A-C
131-11R	Uninfected			N/A	N/A		N/A	N/A	1A-C
131-11R	Uninfected			N/A	N/A		N/A	N/A	1A-C
176-11R	Uninfected			N/A	N/A		N/A	N/A	1A-C
187-11R	Uninfected			N/A	N/A		N/A	N/A	1A-C
189-11R	Uninfected			N/A	N/A		N/A	N/A	1A-C
202-11R	Uninfected			N/A	N/A		N/A	N/A	1A-C
315-11R	Uninfected			N/A	N/A		N/A	N/A	1A-C
