# Supplementary figures and images for: The role of CD101-expressing CD4 T cells in HIV/SIV pathogenesis and persistence
Source: PLoS Pathog. 2022 Jul 22;18(7):e1010723. doi: 10.1371/journal.ppat.1010723 (PMC9348691; doi:10.1371/journal.ppat.1010723)

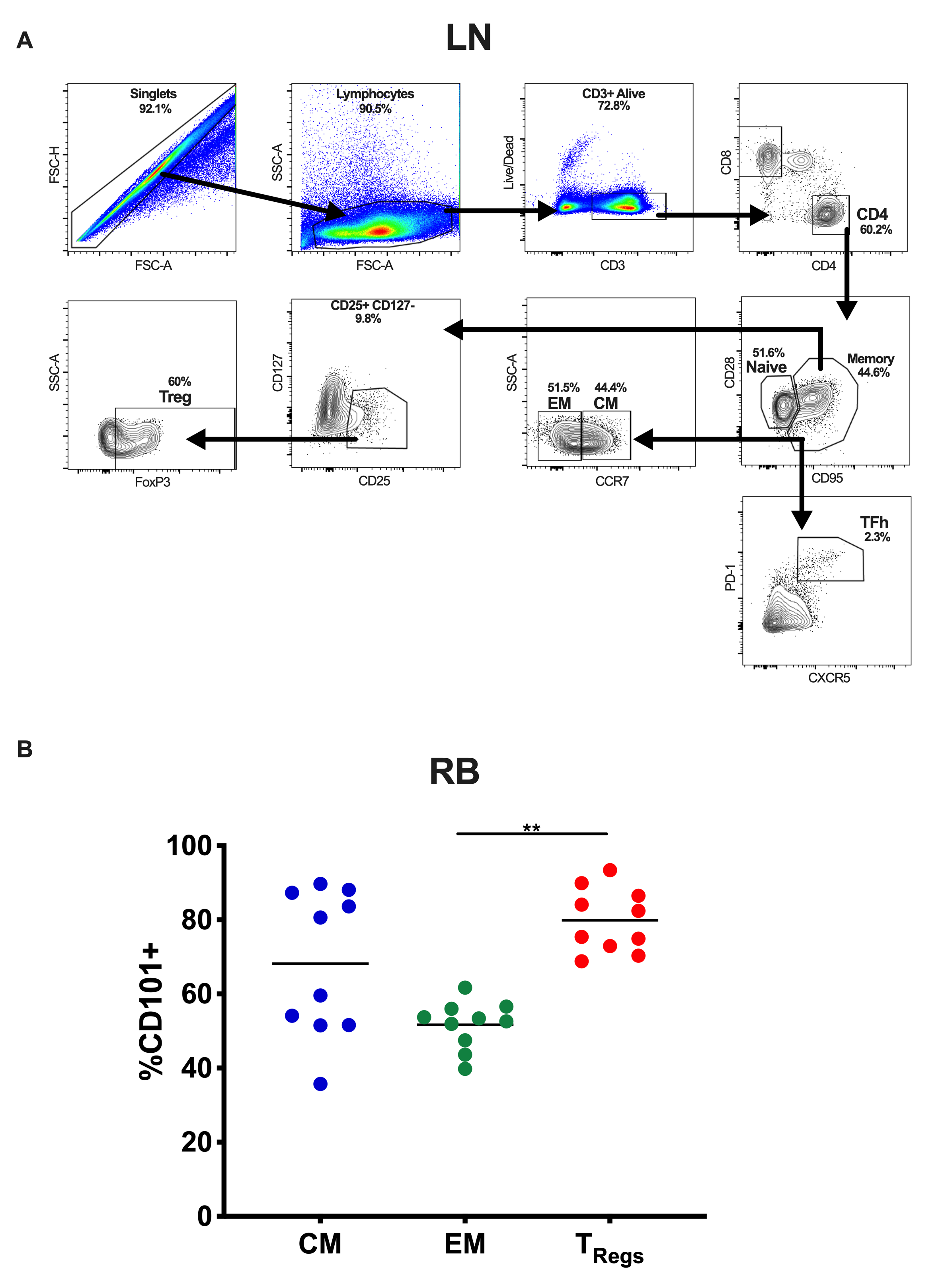

Supplement: S1 Fig — A) Representative gating strategy for CD4 subsets within LN samples from healthy rhesus macaques. B) CD101 expression on CD4 subsets in rectal biopsy samples from healthy rhesus macaques. Lines designate means. (TIFF) [file ppat.1010723.s004.tiff]

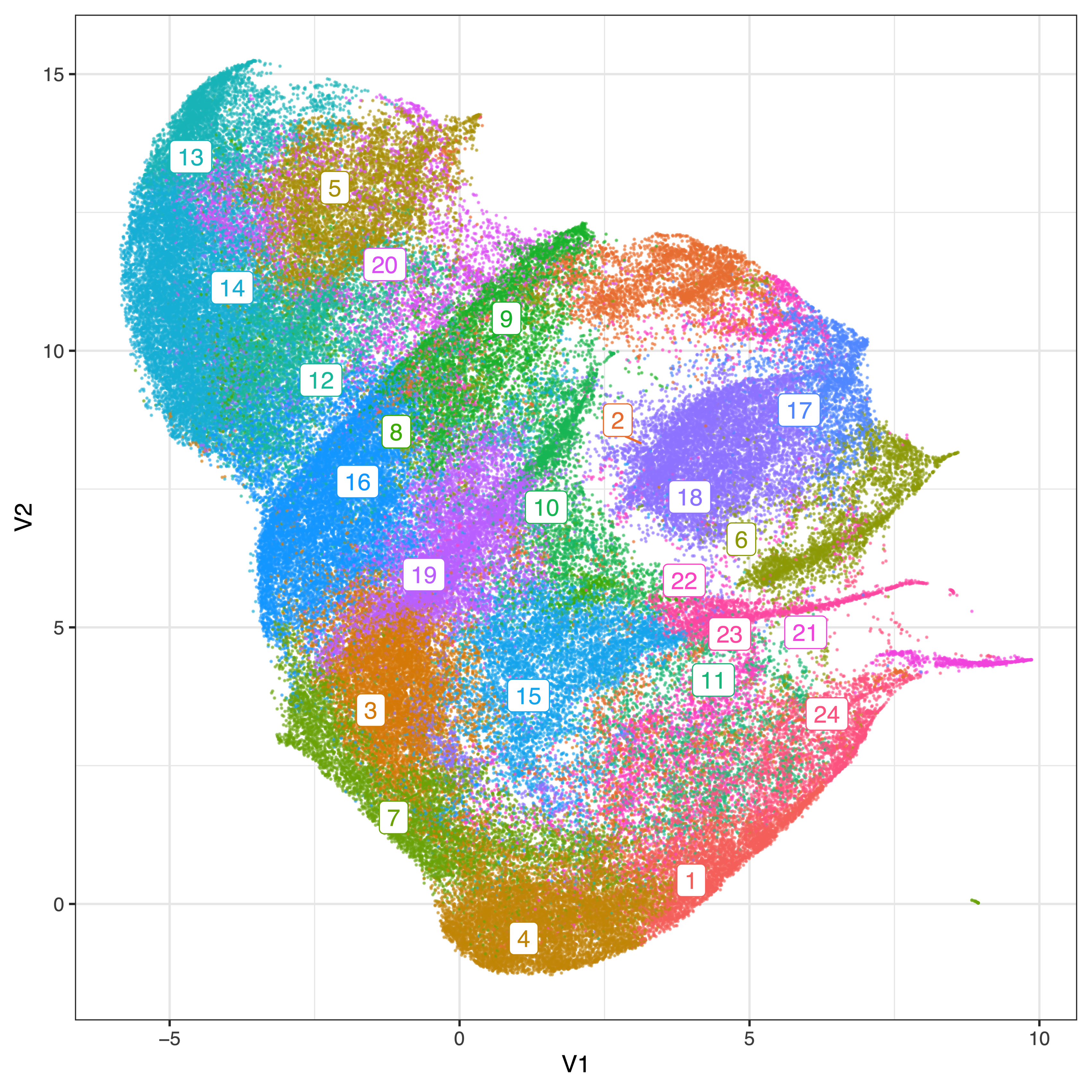

Supplement: S2 Fig — (TIFF) [file ppat.1010723.s005.tiff]

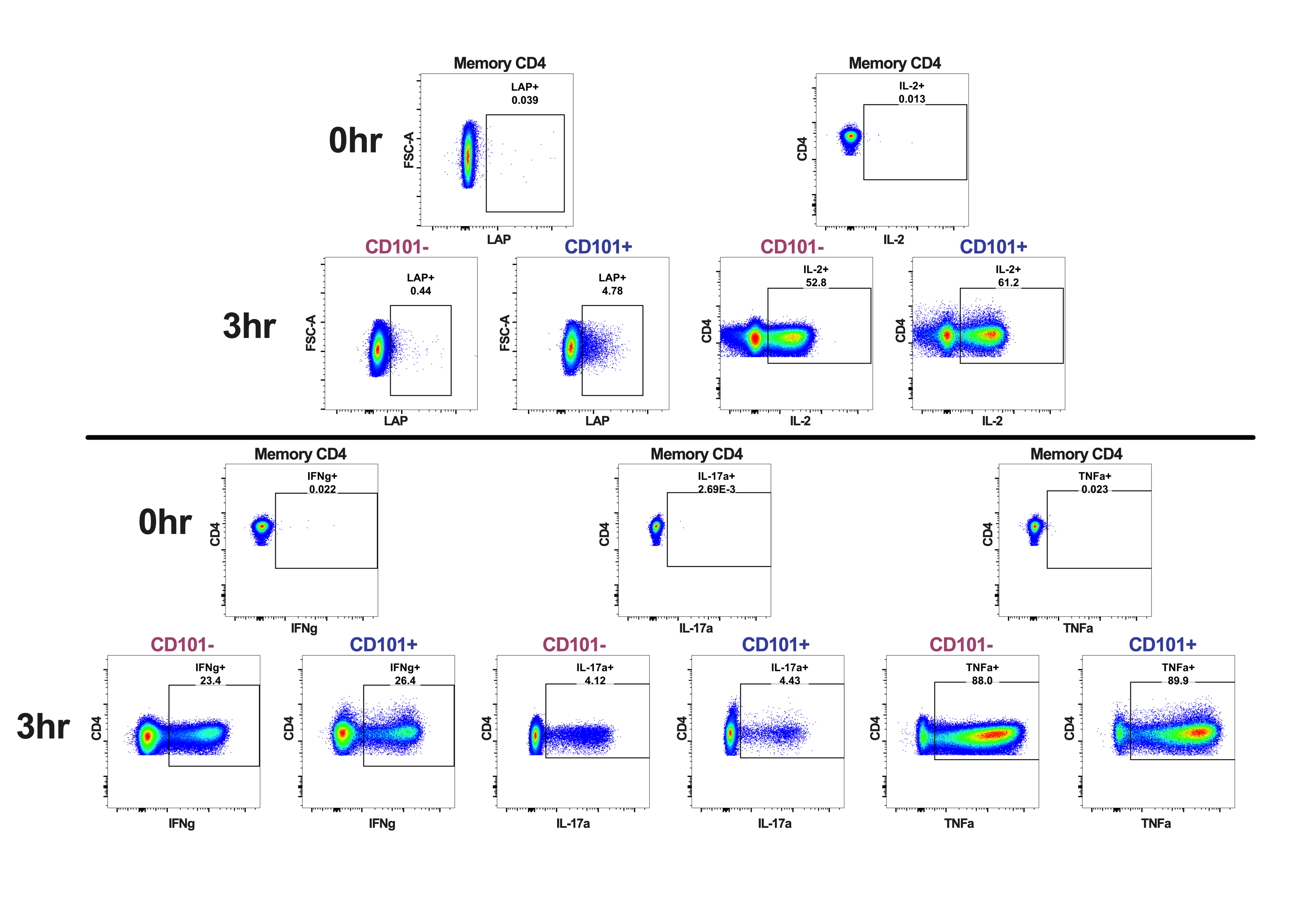

Supplement: S3 Fig — Representative gates for cytokine levels within memory CD4 at 0hr post-stimulation and within CD101- and CD101+ memory CD4 at 3hr post-stimulation with PMA/ionomycin. (TIFF) [file ppat.1010723.s006.tiff]

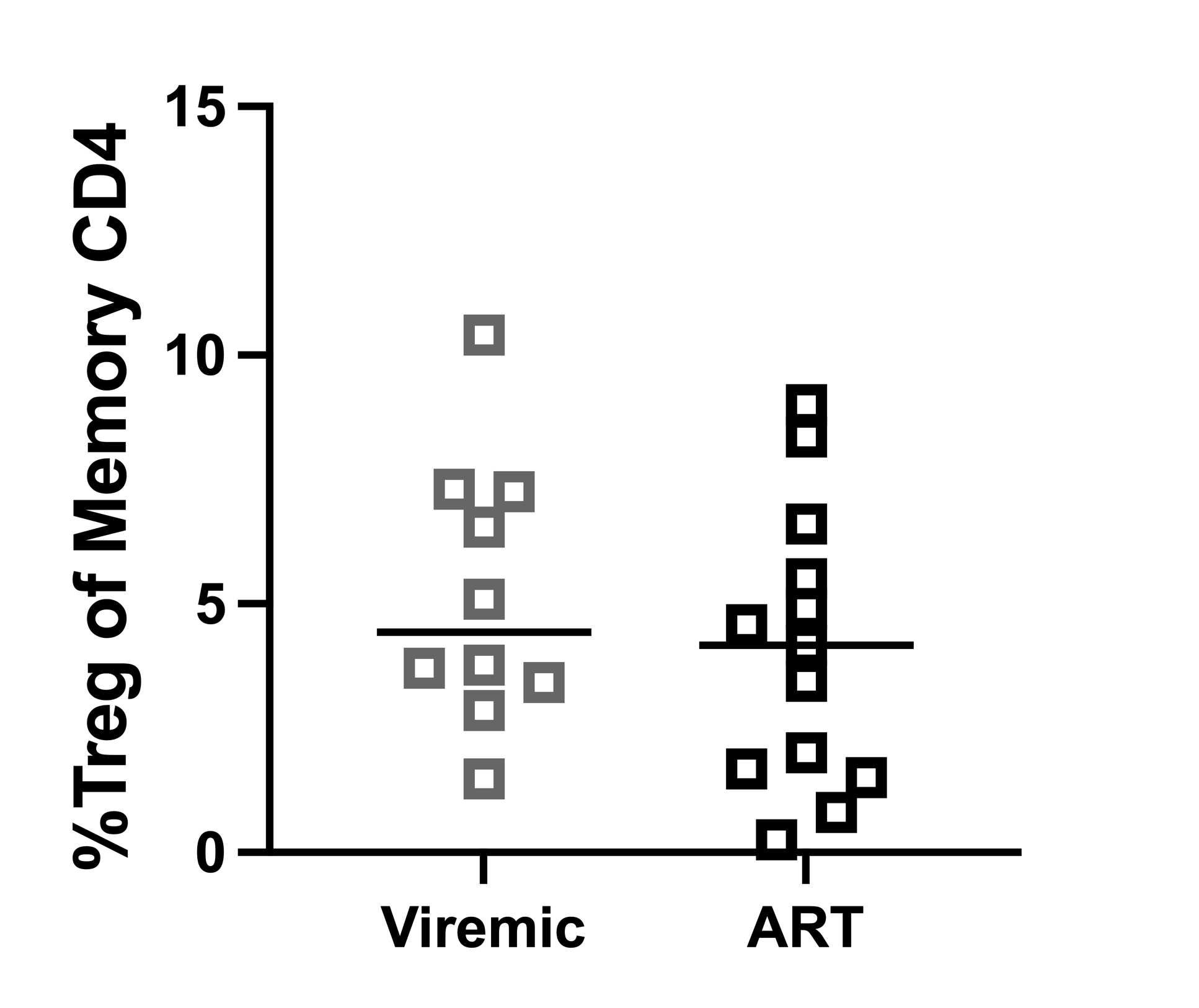

Supplement: S4 Fig — The frequency of Tregs (CD25+ CD127- FoxP3+) within the memory pool of CD4 Tregs evaluated in PBMC from viremic and ART-suppressed individuals. Lines designate means. (TIFF) [file ppat.1010723.s007.tiff]

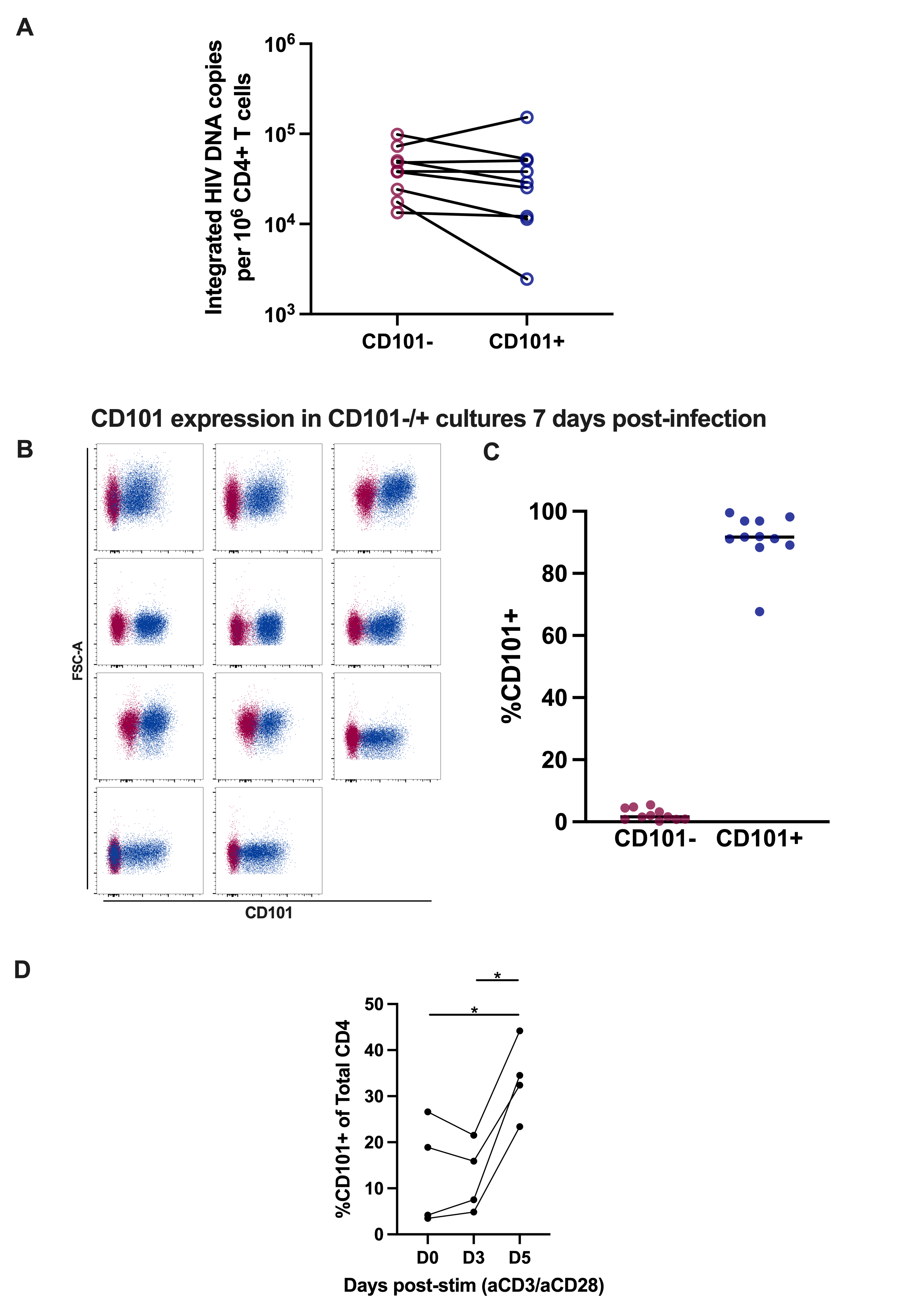

Supplement: S5 Fig — A) Levels of integrated HIV DNA from CD101- or CD101-positive cell cultures 7 days after in vitro infection of sorted cells. B) Overlayed flow plot of expression of CD101 on CD4 T cells from CD101- (maroon) and CD101+ (blue) cultures 7 days after in vitro infection of sorted cells. C) Gated expression levels of CD101 on CD4 T cells from CD101- and CD101+ cultures. D) CD101 expression levels on total CD4 T cells after aCD3/aCD28 stimulation of PBMC from healthy individuals. (TIFF) [file ppat.1010723.s008.tiff]

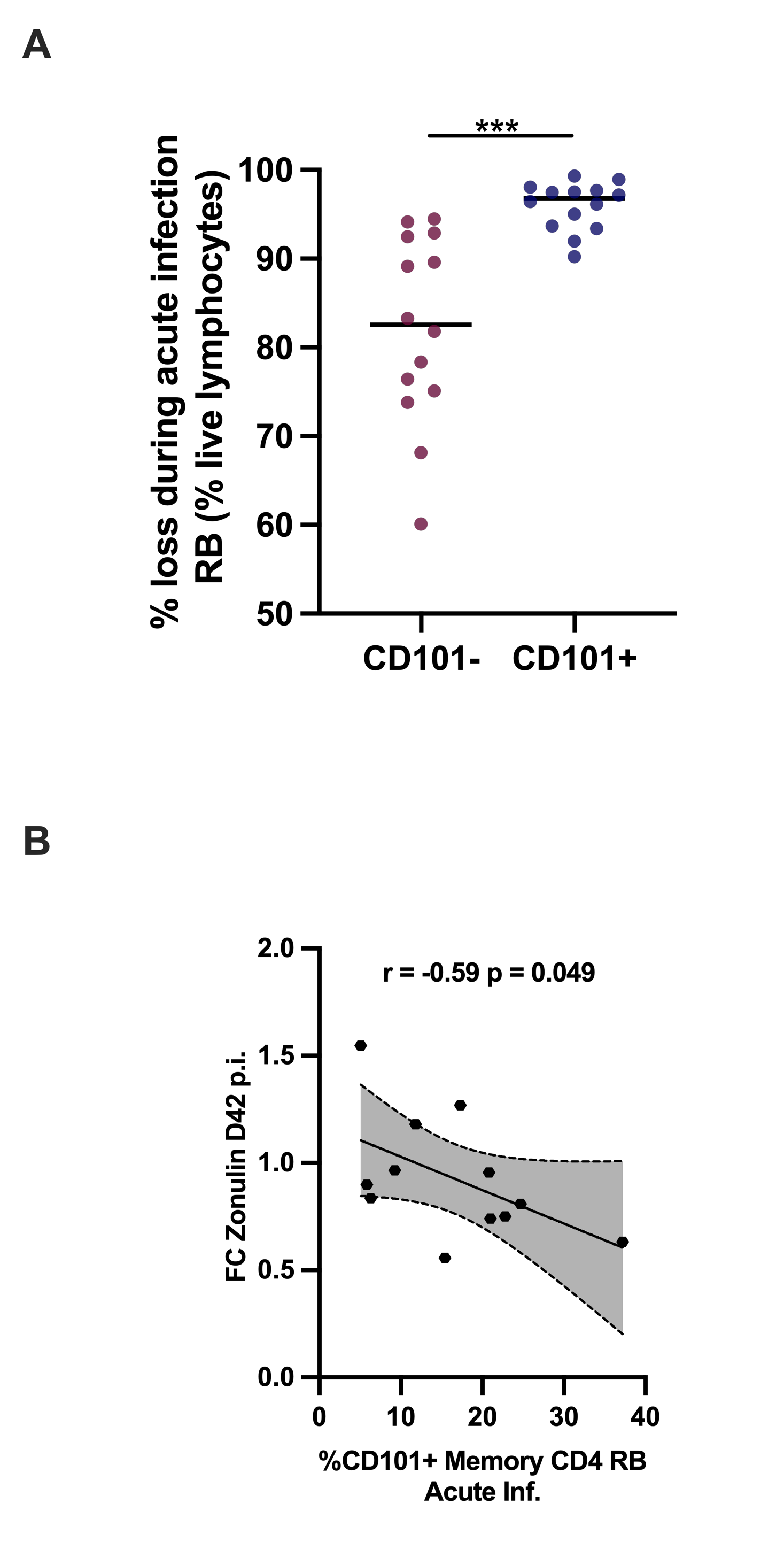

Supplement: S6 Fig — A) Loss of CD101- and CD101+ CD4 T cells in the gut during acute infection, calculated as %loss from baseline using the frequency of CD101- and CD101+ cells of total live lymphocytes. B) Association between levels of CD101-expressing CD4 T cells in the gut during acute infection and fold change of circulating zonulin in plasma at day 42 p.i. compared with pre-infection (spearman correlation with linear regression and 95% confidence interval). (TIFF) [file ppat.1010723.s009.tiff]
